# Supplementary material for: Soybean-Based Polyol as a Substitute of Fossil-Based Polyol on the Synthesis of Thermoplastic Polyurethanes: The Effect of Its Content on Morphological and Physicochemical Properties
Source: Polymers (Basel). 2023 Oct 6;15(19):4010. doi: 10.3390/polym15194010 (PMC10574837; doi:10.3390/polym15194010)
Supplement: Supplementary file 1 [file polymers-15-04010-s001.zip › polymers-2636475-supplementary.pdf]

This supplemental file shows FTIR, DSC, SAXS and DMA data. In Figure S1. FTIR spectra in the region between 4000-500 cm^{-1} are presented. After the polymerization reaction, the NCO band at 2277 cm^{-1} was not noticed. This allows inferring that the polymerization was complete and occurred regardless of the TPU formulation. Subsequent bands characteristic of complete polymerization of urethane group formation and triglyceride bands were observed in all TPUs. The band at 1735 cm^{-1} is attributed to the stretching mode of the carbonyl ester group (CO) of soybean oil. Aliphatic hydrocarbon groups in the flexible domain are visible at 1463, 2922 and 2855 cm^{-1}, while CH_2 bending vibrations appear at 722 cm^{-1} and double bonds at 3010 and 1640 cm^{-1}. The band at 1217 cm-1 corresponds to the stretching mode of the carbonyl ester (CO O) in the single bond (CO) configuration, probably arising from glycerol (COH) groups. Consecutive bands at 1307, 1500 and 1520 cm^{-1} are attributed to the CN, CH_2 CH_3 and NH_2 stretch modes. The band at 3318 cm^{-1} is attributed to the free NH stretching vibration of the urethane groups. The main band contributing to the formation of inter-urethane hydrogen bonds appears in the carbonyl region (1600–1800 cm^{-1}).

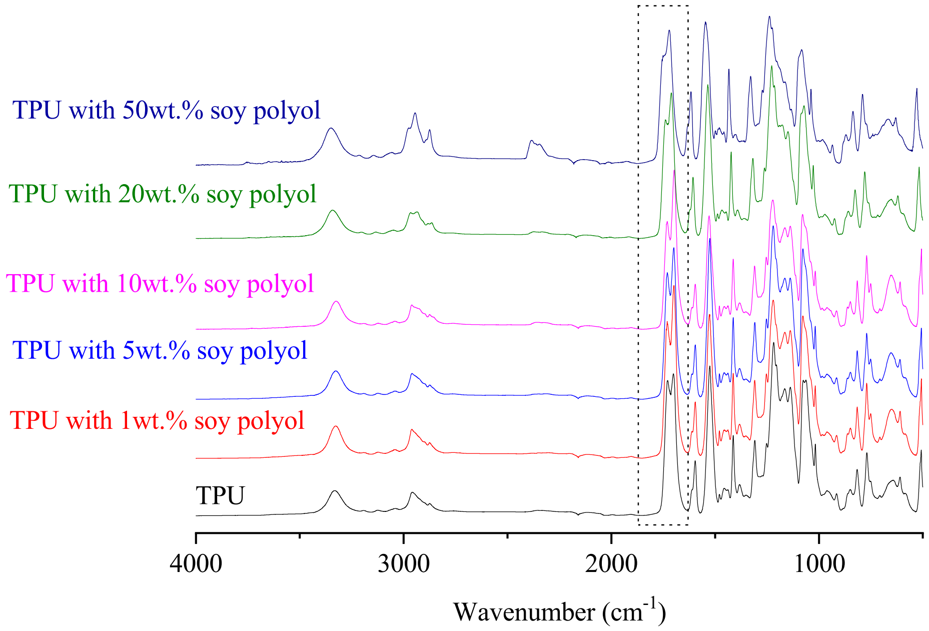

Figure s1. FTIR spectra for TPUs

Figure S2 (a) and S2 (b) show two examples of deconvolutions of FTIR data in the carbonyl region for TPU and TPU samples with 50 wt.% soy polyol. This information was used to estimate the fraction of hydrogen bonds and other characteristics of the system applying equations 1-5. As it can be seen, the urethane bond band associated with hydrogen bonds is strongly affected.

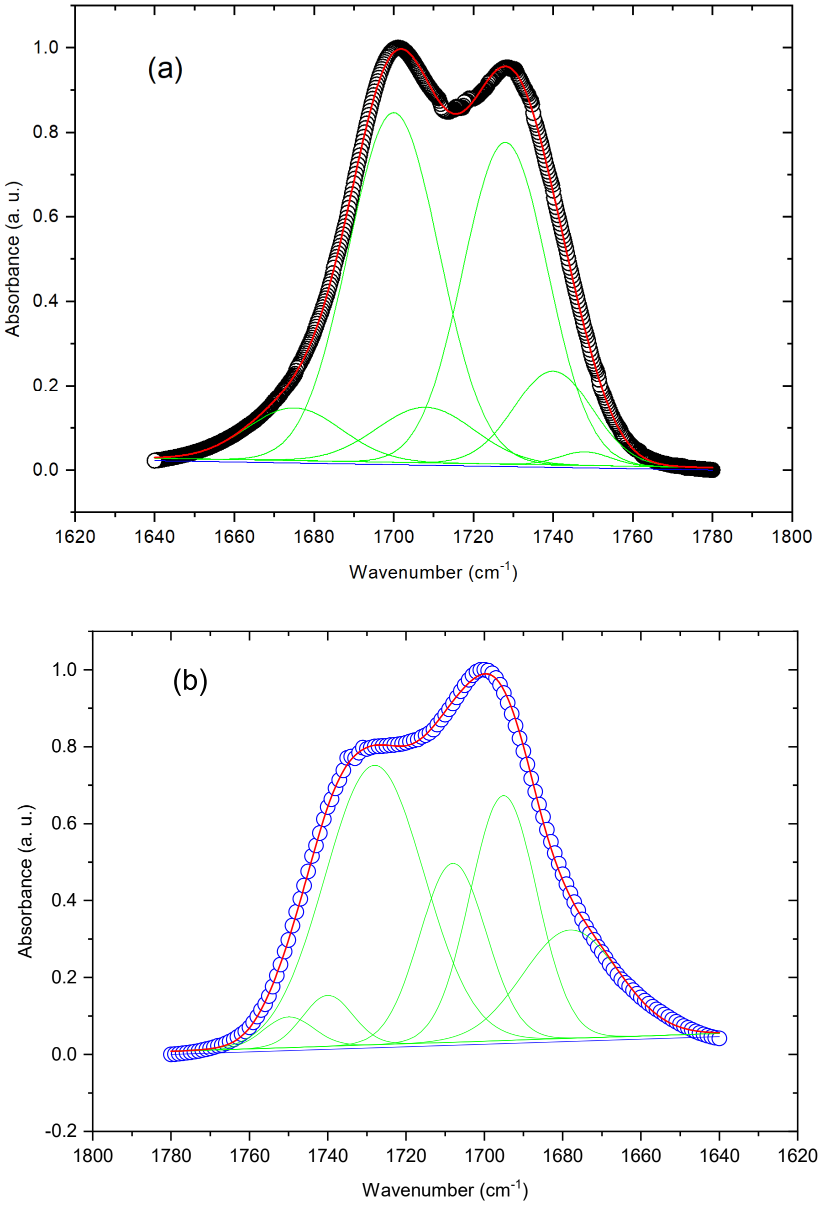

Figure S2. Mathematical deconvolution of spectra in the carbonyl region. (a) TPU and (b) TPU with 50wt.% of soybean polyol.

In Figure S3, the glass transition temperature data measured by DSC are presented. As can be seen, the addition of soy polyol resulted in an increase in this transition due to the formation of crosslinks.

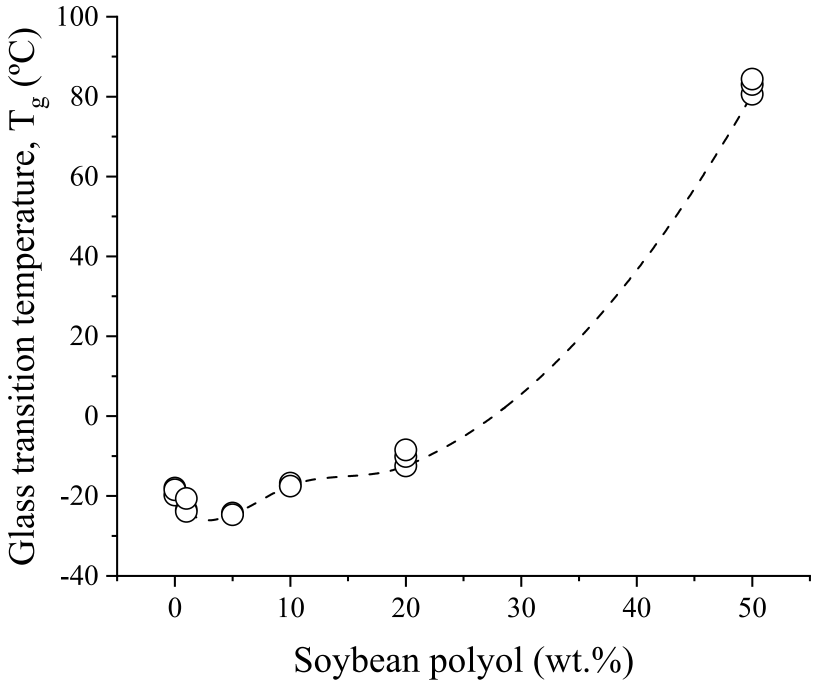

Figure S3. Glass transition temperature as function of soybean polyol content.

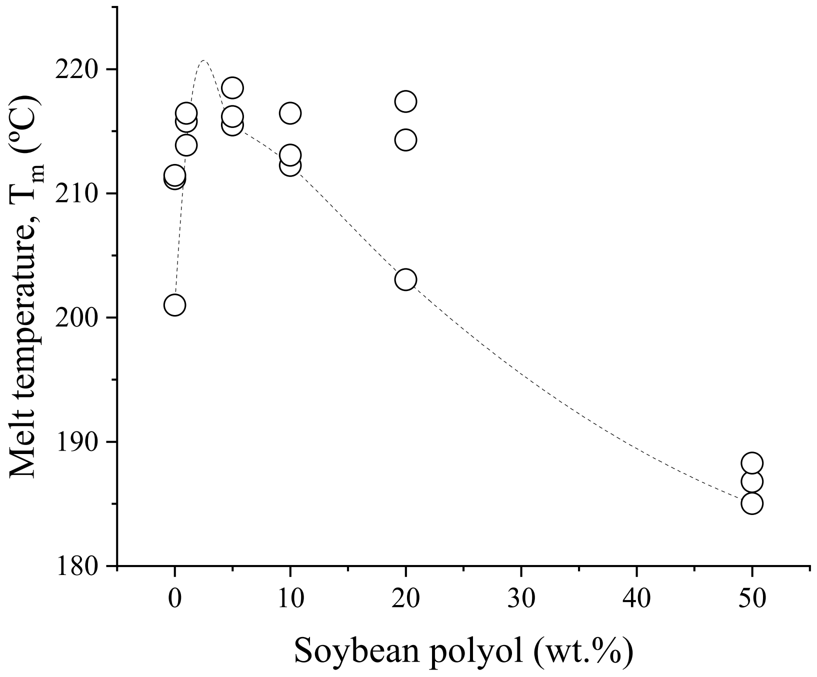

Figure S4. Melt temperature as function of soybean polyol content. The line refers to the spline line trend.

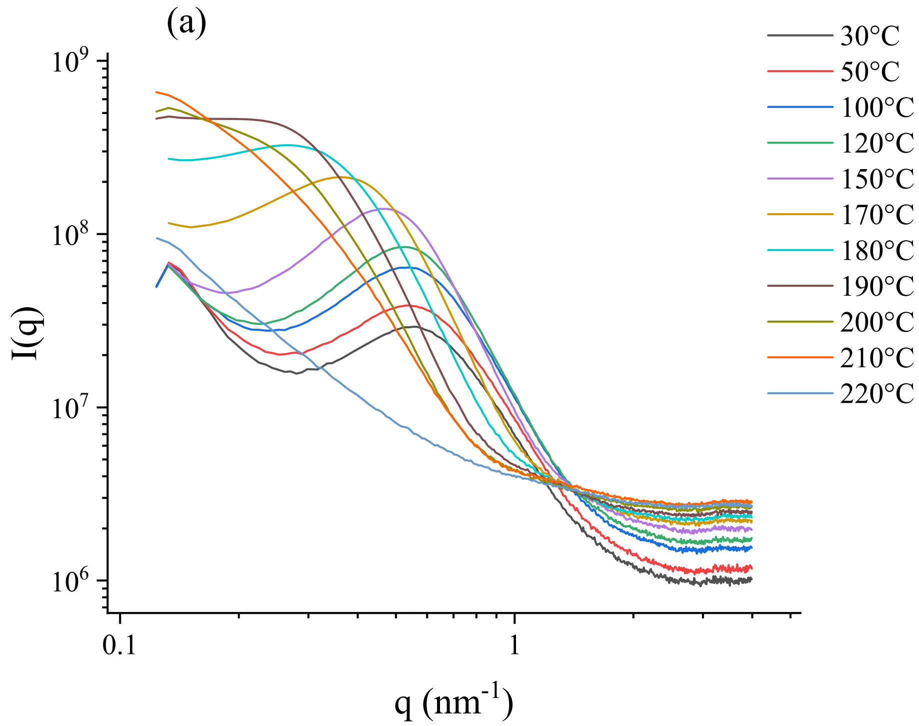

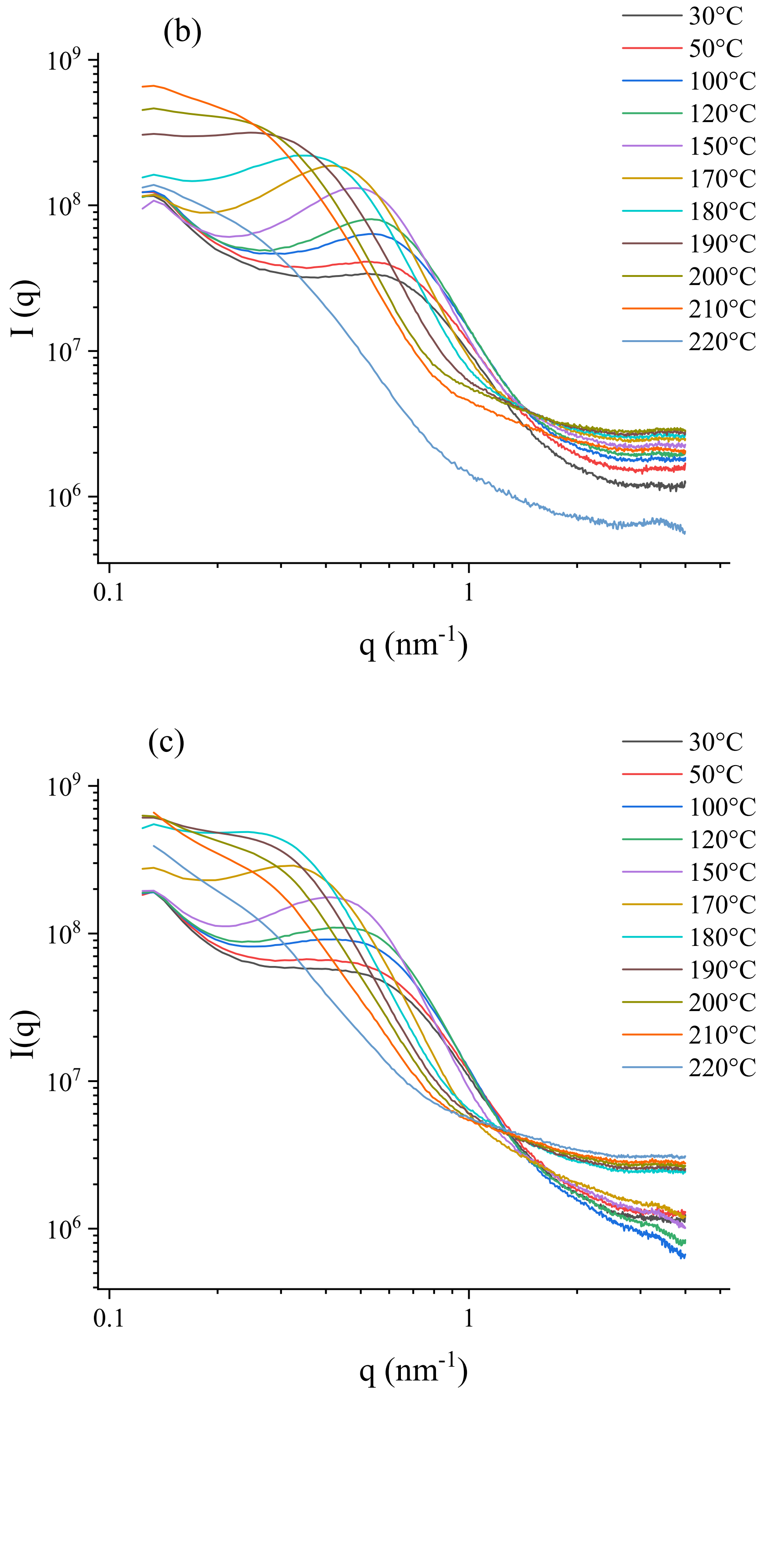
(b)
30°C
50°C
100°C
120°C
150°C
170°C
180°C
190°C
200°C
210°C
220°C
I (q)
10^9
10^8
10^7
10^6
0.1
1
q (nm^{-1})
(c)
30°C
50°C
100°C
120°C
150°C
170°C
180°C
190°C
200°C
210°C
220°C
I(q)
10^9
10^8
10^7
10^6
0.1
1
q (nm^{-1})

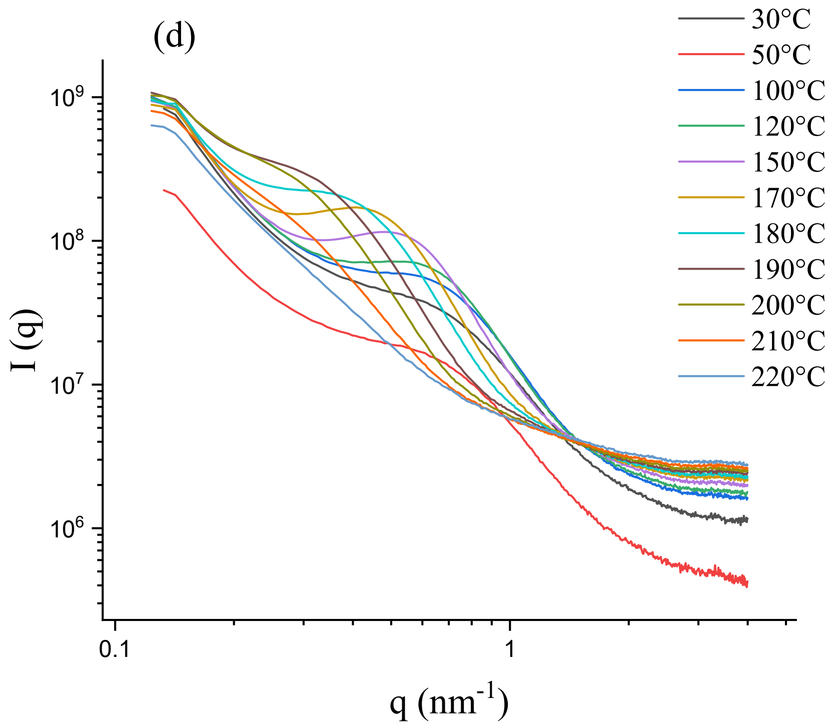

Figure S5. SAXS profiles as a function of temperature: (a) TPU with 1wt.% of soybean polyol, (b) TPU with 5wt.% of soybean polyol, (c) TPU with 10wt.% of soybean polyol and (d) TPU with 20wt.% of soybean polyol.

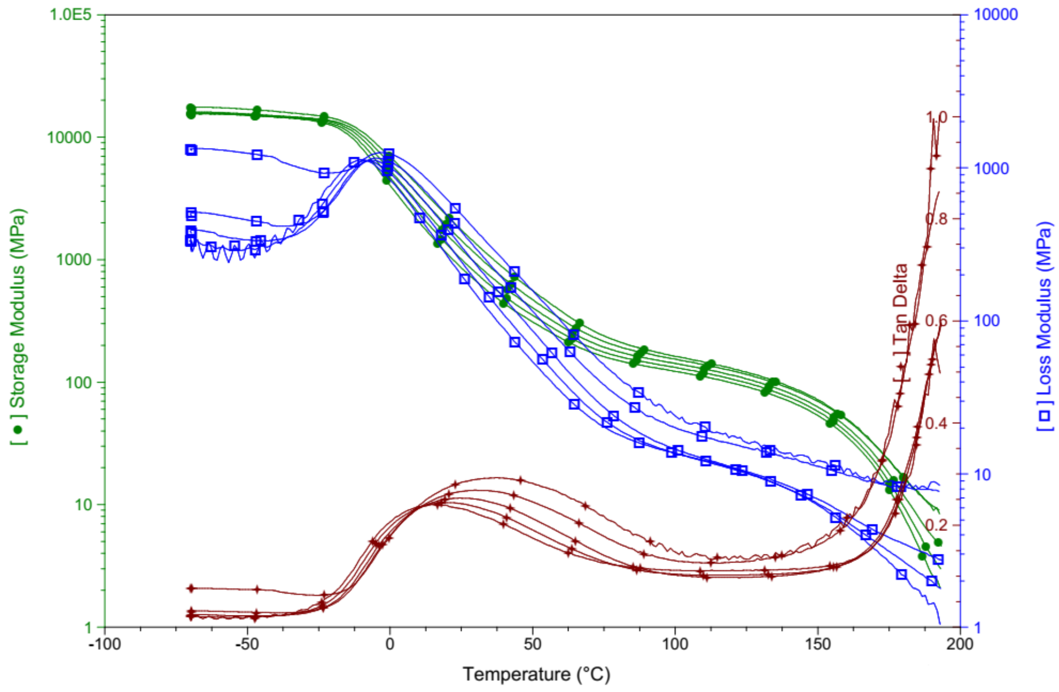

Figure S6. Multi-frequency experiment of TPU sample.

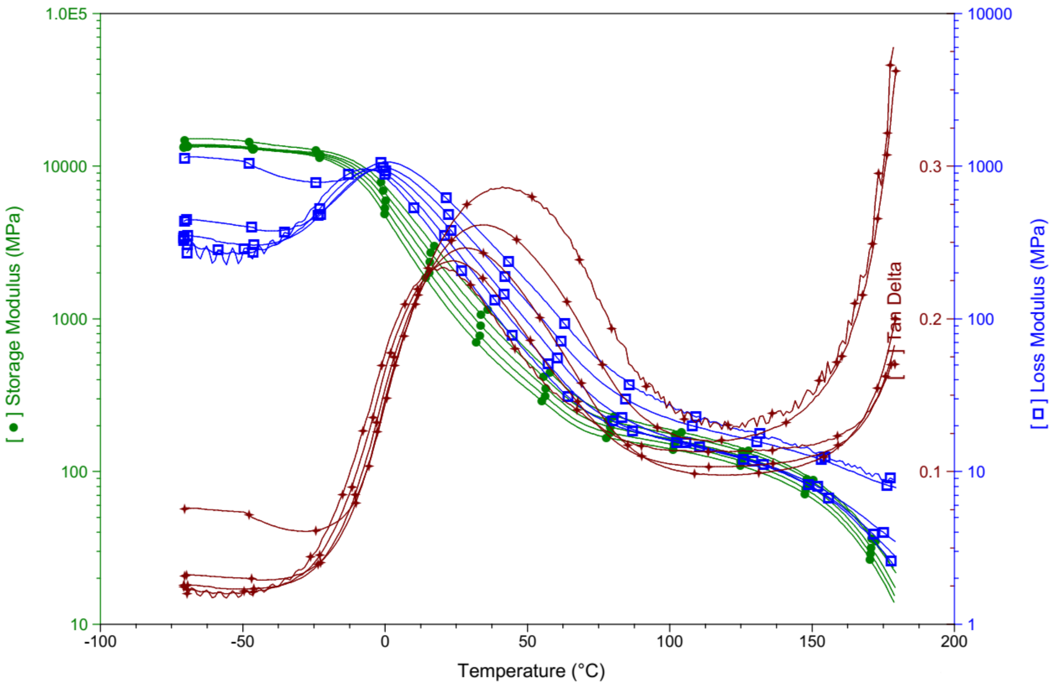

Figure S7. Multi-frequency experiment of TPU with 1wt.% of soybean polyol.

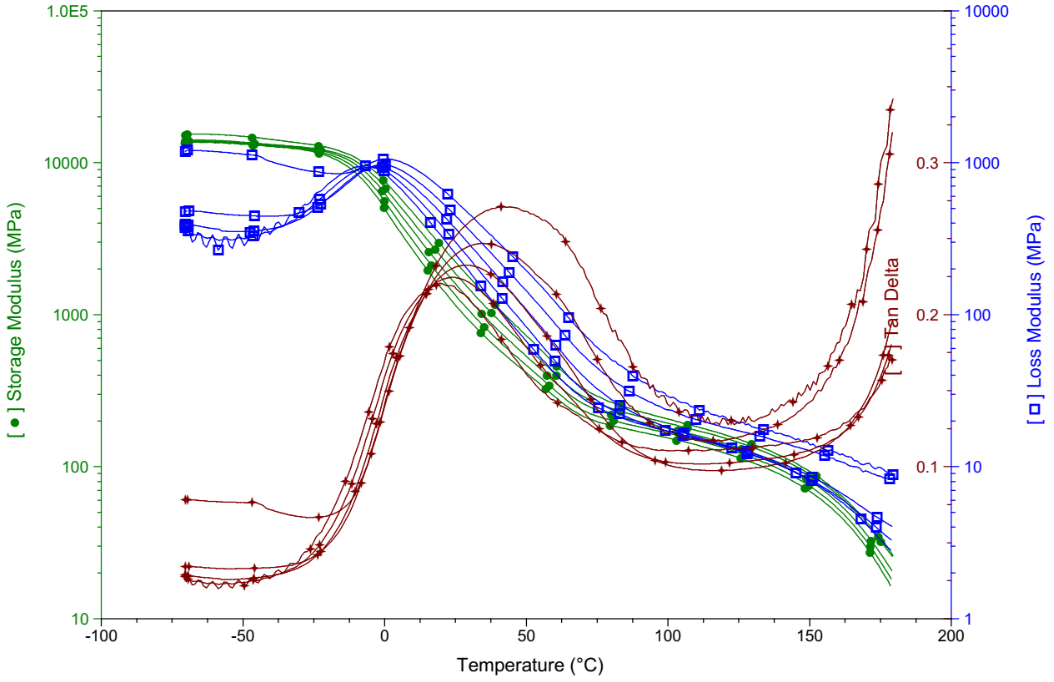

Figure S8. Multi-frequency experiment of TPU with 5wt.% of soybean polyol.

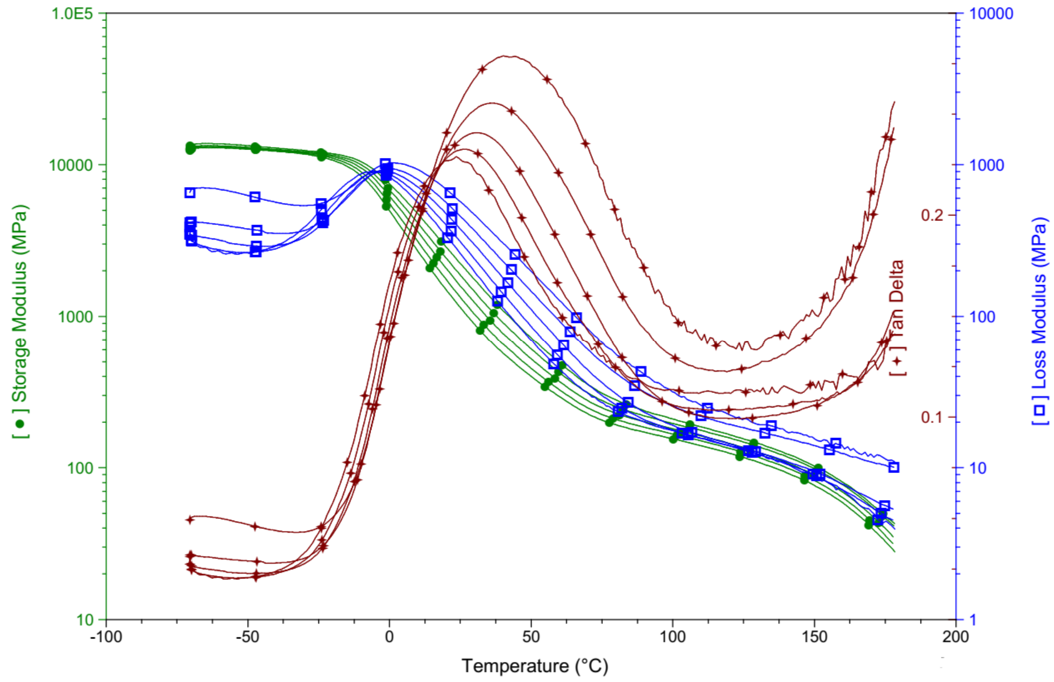

Figure S9. Multi-frequency experiment of TPU with 10wt.% of soybean polyol.

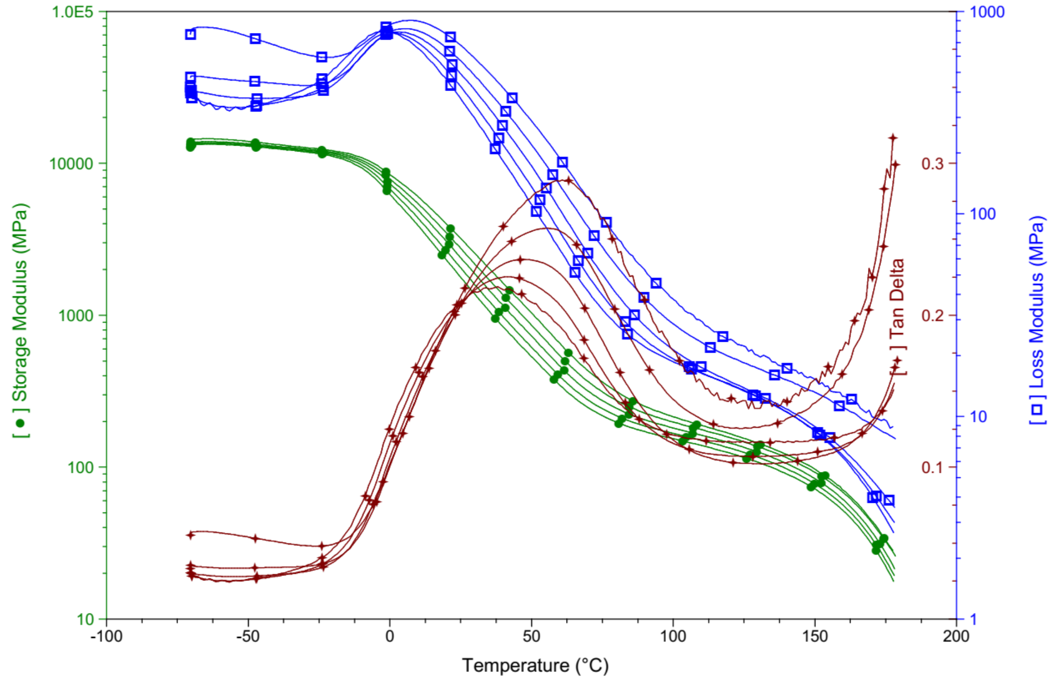

Figure S10. Multi-frequency experiment of TPU with 20wt.% of soybean polyol.

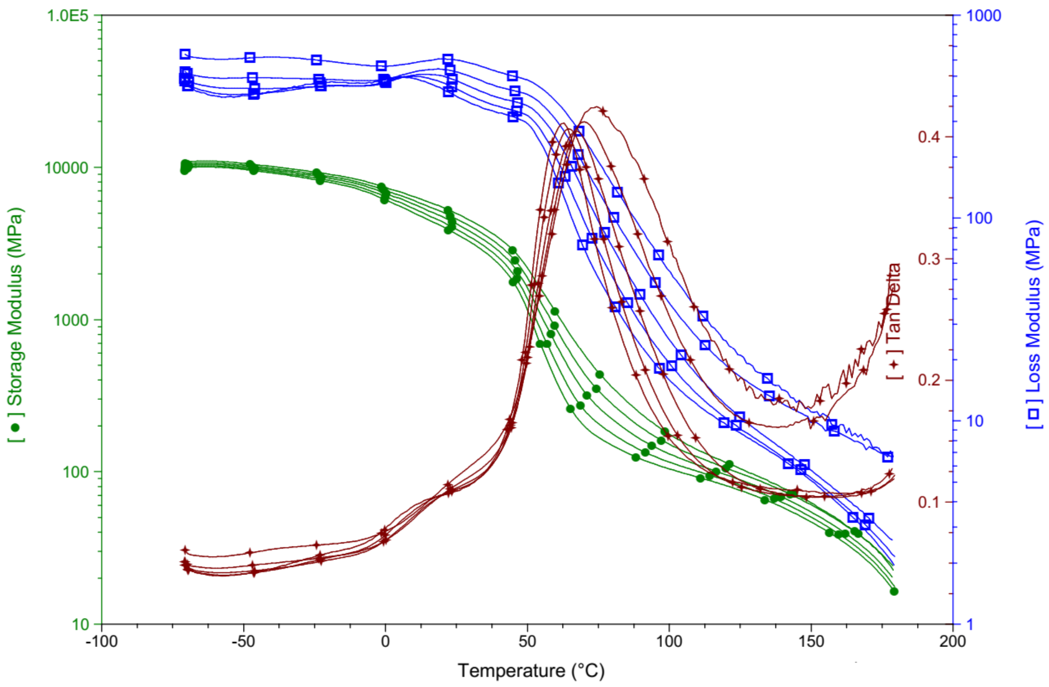

Figure S11. Multi-frequency experiment of TPU with 50wt.% of soybean polyol.

Figure S12 shows the fitted modulus data in the elastic plateau region as a function of frequency. The data were fitted with an exponential function, $E_e' = E_{e\,0}' A exp(R_0 . f)$. Where A and R are constants. Fit data is shown in Table S1. All adjustments had a correlation coefficient greater than 0.9.

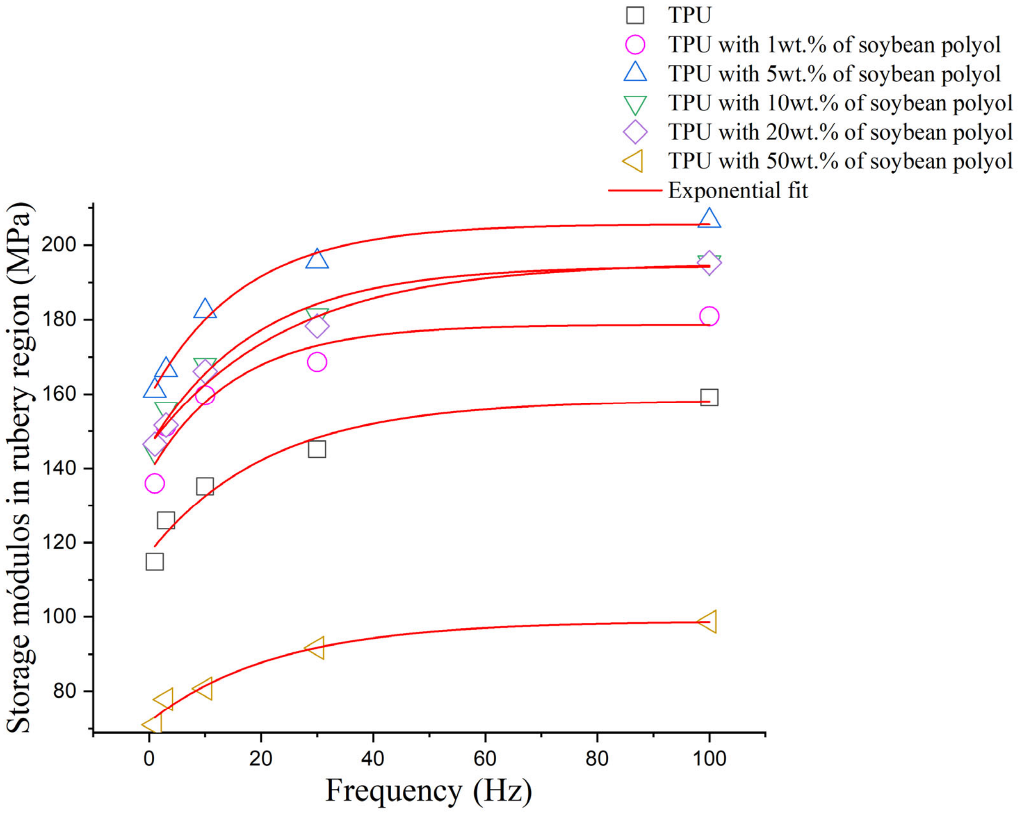

Figure S12. Exponential fit of storage modulus in plateau region, Ee' as function of frequency.

Table S1. Results of exponential fit of storage modulus in *plateau* elastic region.

Soybean polyol (wt.%)	$E_e'_0$ (MPa)	A	R_0	R
0	158.4	-41.2	-0.04	0.959
1	178.7	-40.0	-0.06	0.926
5	205.6	46.6	-0.06	0.991
10	194.4	-48.5	-0.05	0.975
20	195.3	-49.2	-0.04	0.986
50	98.9	-27.1	-0.04	0.976

Table S2. Example of drops used to measure the contact angle in water of the TPU samples.

Soybean polyol (wt.%)	**Image**
0	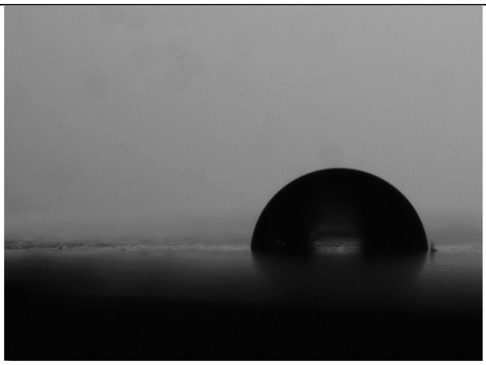
1	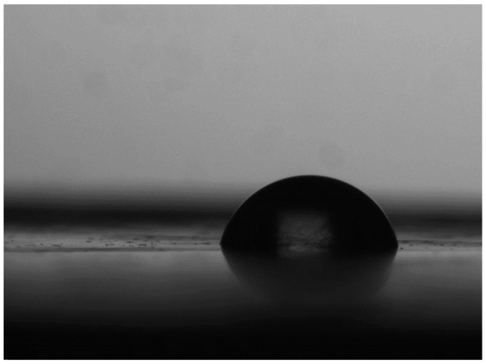
5	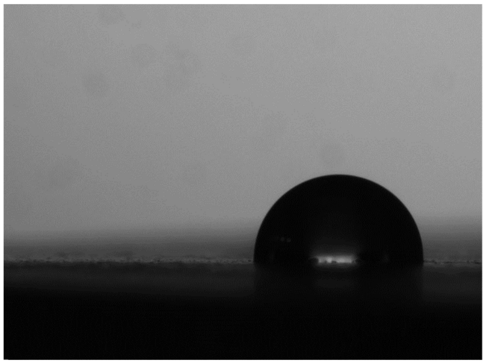
10	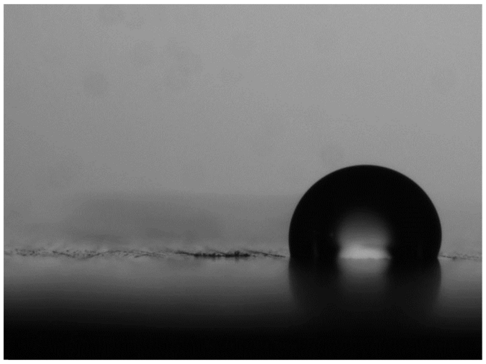

20	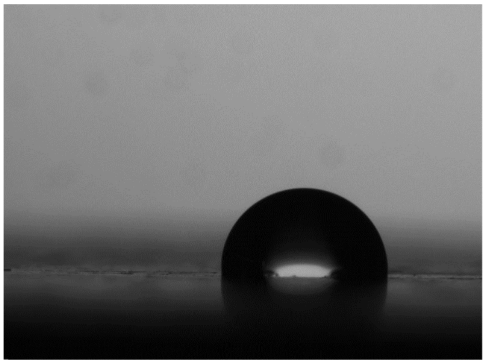
50	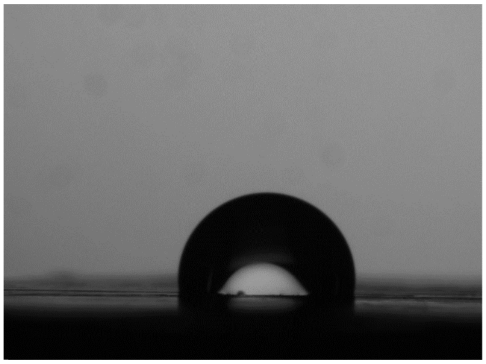

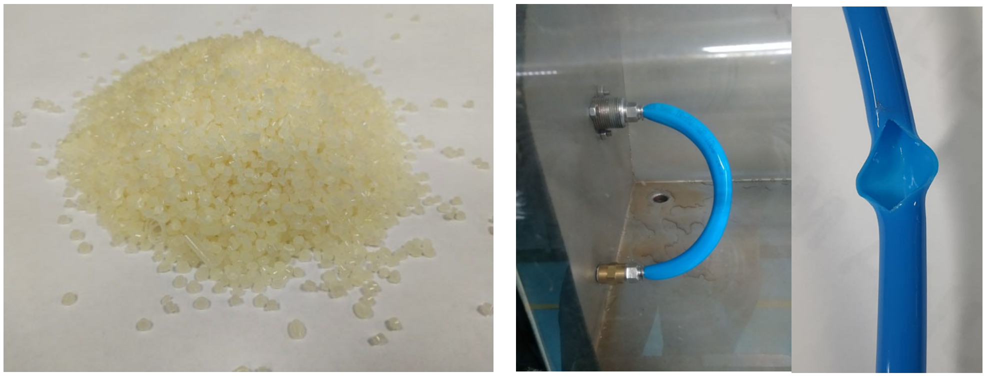

(a) (b)

Figure S13. (a) Polymerized TPU granules. Example of pipes (8x1.25mm) produced after burst test.
